# More on: ‘the role of restraint in fatal excited delirium syndrome’

**DOI:** 10.1007/s12024-023-00722-2

**Published:** 2023-12-12

**Authors:** Hans H. de Boer, Judith Fronczek, Melanie S. Archer

**Affiliations:** Victorian Institute of Forensic Medicine/Dept. of Forensic Medicine, Monash University, 65 Kavanagh Street, Southbank, VIC 3006 Australia

Dear Editor,

We would like to respond briefly to the response of Freeman and Strömmer et al. [1] to our commentary [2] on their paper ‘The role of restraint in fatal excited delirium: a research synthesis and pooled analysis’ [3]. We argued that their paper [3] contains fundamental flaws, which severely undermine their conclusions, and results in a misleading representation of the literature. In their response, the authors express their disagreement with our commentary eloquently, but we believe that their reply does not address what we assert are significant deficiencies in their paper. More specifically, we would like to point to the following:

1. In their reply, the authors argue that much of our critique is misguided and does not relate to the goals and aims of their study. In their introduction, they state that ‘investigating the potential for circular reasoning […] is the primary aim of this paper’ [3]. However, the entirety of the paper shows a much wider scope, and for instance includes statements on mechanism of death and even advice on how to ascribe cause of death. For example, the results in their Tables 2 and 3 are used to support statements such as:
  (I) ‘Restraint [is] the most common factor that is a plausible cause or contributing cause of the death’ [3, p. 684].
  (II) ‘When death has occurred in an aggressively restrained individual who fits the profile of either ExDS [Excited Delirum Syndrome] or AgDS [Agitated Delirium Syndrome], restraint-related asphyxia must be considered a likely cause of the death’ [3, p. 680].
  (III) ‘[The] results provide strong evidence that the more likely it is that a death resulted from restraint, the more likely it is that the death will be attributed to ExDS’ [3, p. 684].
  (IV) ‘There is no evidence to support excited delirium as a cause of death in the absence of restraint’ [3, p. 689].

In our first commentary [2], we explained why we believe that these statements are the result of a flawed interpretation of challenging and limited literature. Statements III and IV come with additional issues:

- For statement III, it should be remembered that Strömmer et al. [3] looked at cases of ExDS/AgDS, and not causes of death. The literature they reviewed shows that the presence of restraint or a diagnosis of ExDS/AgDS should not automatically be equated with the cause of death. In other words: restrained individuals may die for reasons unrelated to the restraint, and the cause of death in cases diagnosed with ExDS or AgDS is not necessarily ExDS or AgDS (see [4–8] for examples). As such, the ‘strong evidence’ [3, p. 684] for a relation between fatal restraint and ExDS as cause of death cannot be inferred from their analysis.
- Statement IV appears to be a true statement in relation to the reviewed literature. However, by its very nature, almost every agitated/delirious person who presents to medical personnel or police officers, will be restrained in some way. This is well-known and was noted by the authors in their introduction [3] and adds little to our understanding of ExDS and related phenomena.

2. Our commentary showed that the diagnosis of the included cases (ExDS, AgDS, or neither) was not always clear from the literature (see Table 1 in [2]), and we questioned the effect this may have had on the results. In their reply, the authors admit that they did not necessarily adopt the diagnoses from the cited literature and classified cases themselves. It is unclear how often this was done, but the author’s response allows for a few examples:
  - In Atherthon et al. [9], no cases were diagnosed with ExDS or AgDS, but in their reply the authors explain that they extracted two cases because it was felt that the term ExDS ‘was relevant’ [1], since excited delirium was mentioned in the key words of the paper and the cases tested positive for a drug with ‘agitation, aggression, violence and hallucinations’ as possible effects. According to the source paper, only one case reportedly displayed ‘erratic and paranoid behaviour’ the evening before he was found dead. The very limited description of the other potentially relevant case did not contain any features commonly associated with excited delirium.
  - Strömmer et al. extracted two non-fatal cases of AgDS from McDaniel et al. [10], apparently since these were described as ‘delusional’ and ‘agitated’ [1]. But the paper of McDaniel et al. describes three cases as delirious [p. 147] and does not mention ExDs or AgDS at all.
  - From O’Halloran and Frank [7], Strömmer et al. extracted 20 fatal cases, apparently since they had ‘descriptions consistent with ExDS/AgDS’ [1], whereas O’Halloran and Frank stated that ‘excited (agitated) delirium’ could be suggested in only 18, without specifying which.
  - The authors’ response [1] provides a revised Table to correct a ‘minor transcription error’, but this new Table raises similar issues with respect to inclusion. For instance, it shows that 49 non-fatal cases of AgDS were extracted from Cole et al. [11], whilst this paper does not use this term. Rather, the 49 individuals in that study were agitated in a general sense only and Cole et al. explicitly state that ‘the assumption cannot be made that all patients in this study had ExDS’ [11, p. 795]. Similarly, Miner et al. [12] apparently provided 68 non-fatal cases of AgDS, but the source paper only describes subsets of generally agitated (*n* = 1146) or delirious (*n* = 260) individuals, with the assumption that many cases in the latter group ‘could have met the various definitions of excited delirium’ [12, p. 365]. No diagnosis of ExDS or AgDS was made.

Strommer et al.﻿ did not mention in their methodology that they would provide diagnoses of ExDS/AgDS themselves based on interpretation of the literature. As such, the statements in their response [1] indicate a far more subjective approach than suggested by the original paper [3], which is problematic without a clear definition of ExDS or AgDS. It is furthermore at odds with their exclusion criteria, which states that cases were excluded if they were ‘not describing case(s) of ExDS/AgDS’ or were describing ‘other delirium’ (Fig. 1. in [3]).

3. In our commentary, we questioned the exclusion of 194 cases of non-fatal, restrained ExDS cases (from [5]). In their response, the authors argue that these cases were not described with sufficient detail to meet the inclusion criteria for the individual case analysis. In a strict sense, this may be true, although there was arguably enough information to use them at least in the all-important analysis of ExDS vs. AgDS deaths associated with restraint/non-restraint. Moreover, in their reply, the authors state that they aimed to ‘create a database of what is in the literature, regardless of presence or absence of details regarding restraint’ [1]. Entirely ignoring such a large quantity of cases is surprising given this aim. The inclusion of these 194 cases (at least in the group data analysis) may have impacted the results on the associations between the presence of restraint, ExDS/AgDS diagnosis, and death.
4. The above issues with inclusion and exclusion stand in addition to the problems with the quality and inherent biases of the literature we previously discussed (see [2]), and strengthen our belief that Strömmer et al.’s study [3] is flawed. In their response, the authors rightfully state that their analysis, like every literature review, was constrained by the available data. However, they are responsible for the design and execution of the study, and the interpretation of the results. In assessing the state of the literature, they could have reconsidered the study or interpreted their data more carefully. Rather, the authors state that they discussed the limitations of the literature ‘appropriately and thoroughly’ [1]. We respectfully disagree with this statement. Mentioning limitations does not mitigate them, and we believe that in a general sense authors should not only mention limitations, but also try to explain their (potential) effect on the results of their analysis, or perhaps even reframe the analysis if the source literature will not allow an accurate conclusion. But their paper [3] does not explain how the differences between published AgDS and ExDS cases (e.g., in reporting of type and duration of restraint, in number of fatal cases) likely affected the results. Instead, Strömmer et al. presented their discussion and conclusions with what appears to be misplaced confidence, implying that the issues with the literature were only minor.
5. In our commentary, we expressed our concern with the statement that ‘when death has occured in an aggressively restrained individual who fits the profile of either ExDS or AgDS, restraint-related asphyxia must be considered a likely cause of the death’ [3, p. 680]. In their reply, the authors restate this opinion and explain their reasoning in more detail. Apparently, ‘the monotonic increased risk of death with more aggressive restraint’ [1] shown in their data forms part of the justification. We hope that by now the reader sees that due to the limitations of the literature, this demonstrated correlation is not necessarily valid. The statement seems also based on the author’s assertion that ‘on the one hand, there are no known pathological mechanisms linking […] ExDS to sudden death, [whilst] there is strong evidence that weighted prone restraint is associated with […] a risk of asphyxia’ [1]. But examining the cause of death of a restrained, delirious individual is not a zero-sum game of choosing between AgDS/ExDS and restraint-related asphyxia. Therefore, a lower likelihood of ExDS as a cause of death should not automatically increase the likelihood of a death due to restraint. In our opinion, the blanket suggestion that a death is ‘likely’ restraint-related when a delirious and (aggressively) restrained individual dies, is as unhelpful as the similar blanket suggestion that such a death is likely due to ExDS (implying it is unrelated to restraint). Where the latter may help to inappropriately absolve the guilty, the former may help to inappropriately inculpate the innocent. When a death occurs in the context of restraint, an opinion on the cause of death should only be given after a thorough and comprehensive post-mortem examination by a qualified, impartial forensic pathologist.

We agree with the authors that the terms AgDS and ExDS are problematic, and the literature about these terms (and related phenomena) is complex and of variable quality. In our opinion, the concerns regarding their usefulness are legitimate, and we note that the use of ExDS is increasingly discouraged (see also [13–15]). At the same time, these terms and the cases that evoke them often give rise to emotive, polarized, and politically charged discussions. In this situation, the onus is on the academic community to provide a balanced, appropriate interpretation of the current state of knowledge to fellow forensic practitioners, the legal community, and the public. After reading the reply of the authors [1], we maintain that the paper by Strömmer et al. [3] fails to do so and does not meet our benchmark of good science.

We thank the editors of *Forensic, Science, Medicine and Pathology* for the opportunity to discuss our concerns, and hope that our contributions are helpful for those who are interested in this complex and important subject.

## Data Availability

The data supporting the findings of this study are available within the article and its references.
